# Statins activate the canonical hedgehog-signaling and aggravate non-cirrhotic portal hypertension, but inhibit the non-canonical hedgehog signaling and cirrhotic portal hypertension

**DOI:** 10.1038/srep14573

**Published:** 2015-09-28

**Authors:** Frank E. Uschner, Ganesh Ranabhat, Steve S. Choi, Michaela Granzow, Sabine Klein, Robert Schierwagen, Esther Raskopf, Sebastian Gautsch, Peter F. M. van der Ven, Dieter O. Fürst, Christian P. Strassburg, Tilman Sauerbruch, Anna Mae Diehl, Jonel Trebicka

**Affiliations:** 1 Department of Internal Medicine I, University of Bonn, Germany; 2 Division of Gastroenterology, Department of Medicine, Duke University Medical Center, Durham, North Carolina, USA; 3 Institute for Cell Biology, University of Bonn, Germany

## Abstract

Liver cirrhosis but also portal vein obstruction cause portal hypertension (PHT) and angiogenesis. This study investigated the differences of angiogenesis in cirrhotic and non-cirrhotic PHT with special emphasis on the canonical (Shh/Gli) and non-canonical (Shh/RhoA) hedgehog pathway. Cirrhotic (bile duct ligation/BDL; CCl_4_ intoxication) and non-cirrhotic (partial portal vein ligation/PPVL) rats received either atorvastatin (15 mg/kg; 7d) or control chow before sacrifice. Invasive hemodynamic measurement and Matrigel implantation assessed angiogenesis *in vivo*. Angiogenesis *in vitro* was analysed using migration and tube formation assay. In liver and vessel samples from animals and humans, transcript expression was analyzed using RT-PCR and protein expression using Western blot. Atorvastatin decreased portal pressure, shunt flow and angiogenesis in cirrhosis, whereas atorvastatin increased these parameters in PPVL rats. Non-canonical Hh was upregulated in experimental and human liver cirrhosis and was blunted by atorvastatin. Moreover, atorvastatin blocked the non-canonical Hh-pathway RhoA dependently in activated hepatic steallate cells (HSCs). Interestingly, hepatic and extrahepatic Hh-pathway was enhanced in PPVL rats, which resulted in increased angiogenesis. In summary, *s*tatins caused contrary effects in cirrhotic and non-cirrhotic portal hypertension. Atorvastatin inhibited the non-canonical Hh-pathway and angiogenesis in cirrhosis. In portal vein obstruction, statins enhanced the canonical Hh-pathway and aggravated PHT and angiogenesis.

Chronic liver injury activates hepatic stellate cells (HSCs), which produce collagen and show increased contraction^1^. Fibrosis and increased contraction of contractile cells lead to portal hypertension and complications^2^. Additionally, neo-angiogenesis has been identified as a key mechanism in the progression of liver cirrhosis with portal hypertension^3^. Neo-angiogenesis occurs within the diseased liver and in the splanchnic vascular bed. The extrahepatic angiogenesis further worsens the portal pressure due to higher portal venous inflow and by opening venous collaterals^3^,^4^,^5^. Interestingly, extrahepatic angiogenesis also occurs in absence of cirrhosis, for example due to portal vein obstruction or thrombosis^6^,^7^,^8^. However, there is scant information about the differences between cirrhotic and non-cirrhotic portal hypertensive angiogenesis.

Statins decrease fibrosis and lower portal hypertension in animals and humans mainly by blunting the RhoA/Rho-kinase-pathway in myofibroblastic HSCs^9^,^10^,^11^,^12^,^13^,^14^,^15^,^16^. Interestingly, RhoA/Rho-kinase-pathway seems to also play a role in the non-canonical Hedgehog-signaling (Hh)^17^,^18^. This crosstalk between RhoA/Rho-kinase and Hh-pathway might be mediated by Shh and is Gli-independent (Fig. 1A).

In liver cirrhosis, also canonical Hh-pathway is activated^19^. In this pathway, the cell surface receptor Patched-1 inhibits Smoothened. When Hh ligands bind Patched-1, Smoothened translocates and activates Gli transcription factors. Previous studies described that Hh pathway leads to the progression of liver diseases. Thereby, Gli enhances transcription of downstream target genes, which activate HSCs and promote the survival of these fibrogenic and contractile cells^19^,^20^,^21^,^22^,^23^,^24^,^25^.

Interestingly, it has been described that statin treatment might decrease Hh activation^26^,^27^. However, it is unknown whether statins interfere with canonical or with the non-canonical Hh signaling and which is their role in angiogenesis induced by portal hypertension.

Therefore, we investigated (i) the pathophysiological differences in angiogenesis induced by cirrhotic or non-cirrhotic portal hypertension, (ii) the effects of statins on angiogenesis in cirrhotic and non-cirrhotic portal hypertension and (iii) their role on the canonical and non-canonical Hh-pathway in portal hypertension.

## Results

### Hemodynamic changes in rats with portal hypertension after statin treatment

We compared two animal models of cirrhotic portal hypertension (BDL; CCl_4_-intoxication) with non-cirrhotic portal hypertension (PPVL). One week before sacrifice all animals received either atorvastatin-chow or control-chow (Fig. 2A). Atorvastatin reduced portal pressure in cirrhotic, CCl_4_-intoxicated rats, due to a significantly decreased hepatic vascular resistance compared to untreated animals (Fig. 2B,C). Splanchnic and systemic vascular resistance remained unchanged after atorvastatin treatment (Fig. 2D–F). Moreover, shunting was significantly decreased and accompanied by a slight increase in cardiac output (p = 0.0625) (Fig. 2E–G). By contrast, in non-cirrhotic PPVL rats, portal pressure increased compared to untreated PPVL rats, despite lower hepatic vascular resistance (Fig. 2B,C). This is accompanied by a significant decrease in splanchnic and systemic vascular resistance (Fig. 2D–F). Atorvastatin treatment led to a dramatic augmentation of shunt flow and cardiac output in PPVL rats compared to untreated PPVL rats (Fig. 2E–G).

In summary, portal pressure, hepatic vascular resistance and shunt flow in cirrhosis were decreased by atorvastatin, as previously shown for the BDL model^13^. By contrast, they were enhanced in non-cirrhotic portal hypertension.

### Hh signaling and profibrotic markers in human and rat portal hypertension

Since atorvastatin decreased hepatic vascular resistance in portal hypertension, we compared the expression of Hh-signaling in atorvastatin-treated animals with untreated animals.

It has been shown, that Shh and Gli are increased in cirrhosis, as well as RhoA/Rho-kinase-pathway. Interestingly, in human cirrhotic liver samples Sonic hedgehog (Shh) and Glioma associated oncogen family zink finger-2 (Gli-2) mRNA levels were increased (Fig. 3A). Similarly, Shh and Gli-2 protein expression was significantly upregulated in cirrhotic livers (Fig. 3B,C). Furthermore, the Hh components Shh and Gli-2 were significantly downregulated in cirrhotic livers of BDL and CCl_4_ – intoxicated rats after atorvastatin treatment (Fig. 3D). The mRNA levels of the profibrotic markers α-SMA, collagen-1, as well as vimentin were decreased after atorvastatin treatment in BDL and CCl_4_-intoxicated rat liver samples (Fig. 3E).

By contrast, Shh mRNA levels were significantly increased in PPVL rats (Fig. 3D). Gli-2 mRNA levels were significantly upregulated in atorvastatin treated PPVL rats compared to the control group (Fig. 3D). mRNA levels of Hedgehog interacting protein (Hhip), a Hh-inhibitor, were significantly downregulated in PPVL rats and secreted frizzled-related protein 1 (sFRP1), a downstream target of Gli-2, was highly upregulated after atorvastatin treatment (Fig. 3D). In PPVL rats, the α-SMA, collagen-1 and vimentin levels were not altered by atorvastatin (Fig. 3E). Hh-signaling is enhanced in liver cirrhosis and atorvastatin blunts Hh-pathway together with profibrotic factors. By contrast, in the livers of PPVL rats, atorvastatin enhanced the Hh-pathway, which might be associated with increased levels of Hh ligands into circulation.

### *In vitro* analyses of primary HSCs and human derived LX-2 cells

These cells are crucially involved in fibrosis and PHT. Cultured primary rat HSCs, were incubated with different doses of atorvastatin (10^−4^ M, 10^−5^ M and 10^−6^ M). The rational for using scratch assay to investigate the migration is that the cell-ECM and cell-cell interactions are still intact compared to other methods such as Boyden Chamber^28^. Interestingly, HSC migration as measured by scratch assay was significantly blunted by atorvastatin incubation (Fig. 4A,B). mRNA levels of the profibrotic markers α-SMA and collagen-1 were significantly downregulated after atorvastatin treatment in primary HSCs (Fig. 4C) and atorvastatin induced a significant decrease of Shh and sFRP1 (Fig. 4D).

To differenciate between canonical and non-canonical Hh-signaling, we investigated statin effects on RhoA/Rho-kinase-pathway in activated HSCs. Human derived LX-2 cells transfected with a constitutively active (CA) RhoA plasmid had significantly increased mRNA and protein expression levels of Shh, compared to control cells and cells transfected with dominant negative (Dn) RhoA (Fig. 4E,F), while Gli-2 expression remained unchanged (data not shown). Incubation with cyclopamine, an inhibitor of the canonical Hh-pathway, reversed these effects (Fig. 4E).

The angiogenic and profibrotic potential of activated HSCs, as well as Hh-signaling is blunted by statins. This effect is mainly due to the non-canonical pathway, where Gli-2 expression has not been significantly decreased and Shh protein expression was upregulated by constitutive active RhoA.

### Angiogenesis assays *in vivo*

Since shunt flow was reduced in cirrhotic and enhanced in non-cirrhotic portal hypertensive animals as compared to controls, we investigated the role of angiogenesis in extrahepatic vessels. Matrigels (collagen plug) were implanted for the investigation of angiogenesis in cirrhotic and non-cirrhotic portal hypertension. Intraperitoneal and subcutaneous matrigels were immunohistochemically stained for endothelial cells (ECs) with an antibody against CD31 and for vascular smooth muscle cells (VSMCs) with an α-SMA antibody. Similar to the hemodynamic changes, sprouting of new vessels in matrigel was decreased in cirrhotic portal hypertension (BDL/CCl_4_) by atorvastatin, whereas it was enhanced in non-cirrhotic portal hypertension (PPVL) by atorvastatin treatment (Fig. 5A,D; Suppl. Fig. 1A–D).

Interestingly, atorvastatin also reduced VSMC migration in subcutaneously implanted matrigels (Fig. 5D; Suppl. Fig. 1D), which cannot be explained by shear stress due to portal hypertension in cirrhosis.

By contrast, atorvastatin treatment enhanced intraperitoneal migration of VSMCs (α-SMA) and ECs (CD31) into the matrigel in PPVL rats (Fig. 5A,B; Suppl. Fig. 1A,B). Furthermore, the subcutanous migration of ECs (CD31) inside the matrigel was enhanced in atorvastatin treated PPVL rats but remained unchanged for VSMCs (α-SMA) (Fig. 5C,D; Suppl. Fig. 1C,D). Atorvastatin decreased subcutaneous VSMC migration in Sham rats (Fig. 5D; Suppl. Fig. 1D).

Since, similarly to the activation of Hh-signaling in the respective livers, angiogenesis is blunted in cirrhosis after atorvastatin treatment, but enhanced in non-cirrhotic portal hypertension, we investigated the role of canonical and non-canonical Hh-signaling in the extrahepatic vessels.

### Analysis of extrahepatic vessels

We evaluated the expression of Hh-signaling pathway in hepatic arteries of cirrhotic and healthy humans, as well as in the matrigel of the portal hypertensive animals after treatment with atorvastatin. Non-canonical Hh (Shh) was highly upregulated in cirrhotic hepatic arteries compared to non-cirrhotic arteries (Fig. 6A). In contrast, Gli2 mRNA levels were not affected (Fig. 6A), which might suggest the activation of non-canonical Hh-signaling. The explanted matrigels were analyzed using RT-PCR. Unchanged Gli-2 expression indicated that atorvastatin treatment did not alter canonical Hh-signaling, while downregulation of Shh abolished the non-canonical variant in subcutaneous and intraperitoneal implanted matrigels of BDL and CCl_4_-intoxicated rats (Fig. 6B,C). Interestingly, canonical Hh-pathway (Gli-2) was highly upregulated in PPVL rats treated with atorvastatin in both, intraperitoneal and subcutaneous implanted matrigels (Fig. 6B,C). The migration of VSMC *in vitro* was inhibited by atorvastatin incubation, similarly to the effect of atorvastatin on VSMC migration into subcutaneous matrigel of Sham rats (Fig. 6D). Moreover, the inhibition of canonical Hh-pathway using cyclopamine had no effect on the migration of VSMCs *in vitro.* By contrast, human umbilical vein endothelial cells (HUVECs) formed significantly more tubes after incubation with atorvastatin *in vitro*.

**Figure 1:**
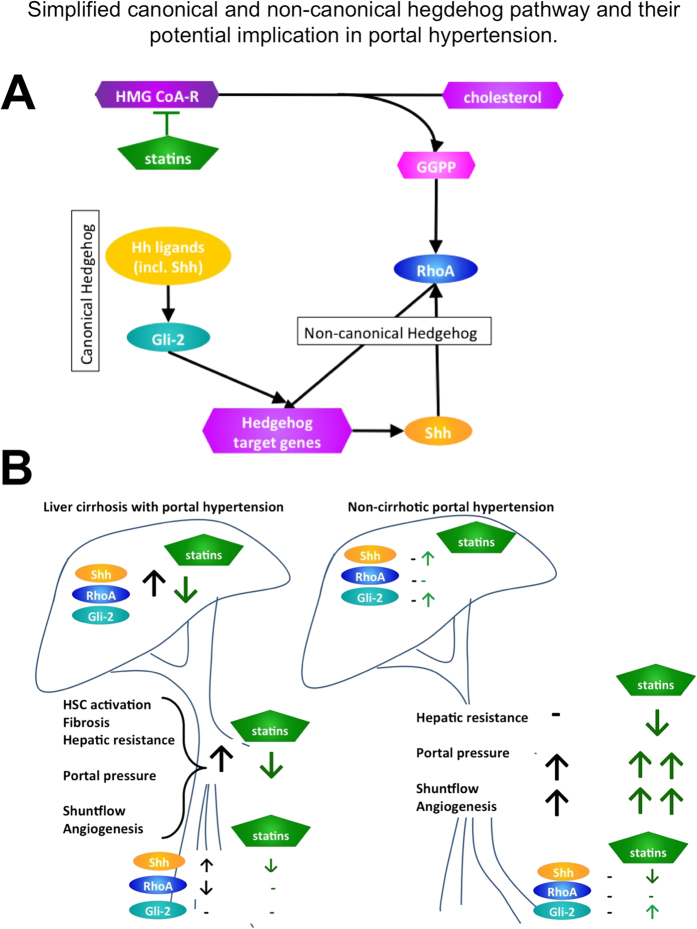
Simplified canonical and non-canonical hegdehog pathway and their potential implication in portal hypertension. (**A**) Statin inhibit RhoA activation by hindering its isoprenylation by depletion of geranylgeranyl-pyrophosphate (GGPP). RhoA seems also to play a role in the non-canonical Hedgehog-signaling (Hh). This crosstalk between RhoA/Rho-kinase and Hh-pathway might be mediated by Shh and is Gli-independent. The canonical Hh-pathway is activated by Hh ligands, which after several steps activates Gli. Gli enhances transcription of downstream target genes. (**B**) Statins decrease fibrosis and lower portal hypertension in animals and humans with liver cirrhosis by blunting the RhoA/Rho-kinase-pathway and downregulation of canonical and non-canonical Hh-pathway in myofibroblastic HSCs. The non-canonical Shh/RhoA-signaling seems to play a major role in the extrahepatic angiogenesis in cirrhosis, whereas Gli-2 is a predominant mediator of canonical Hh-pathway in non-cirrhotic portal hypertension potentially mediating angiogenesis and aggravation of portal hypertension.

**Figure 2:**
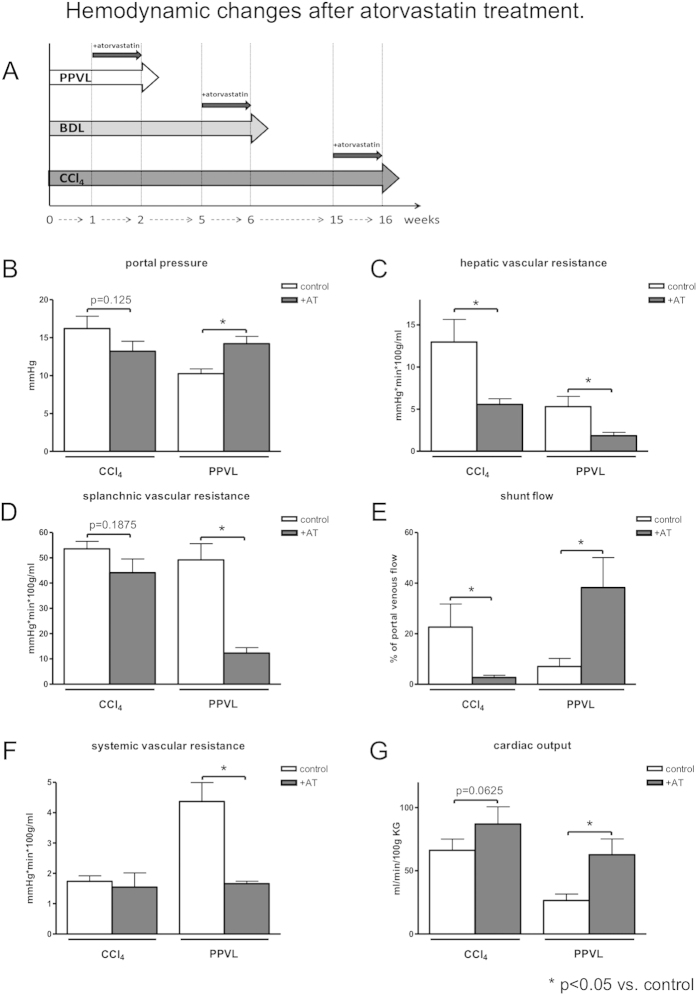
*In vivo* hemodynamic measurements of CCl_4_-intoxicated and partial portal vein ligated (PPVL) rats with and without atorvastatin treatment. (**A**) One week after PPVL, five weeks after BDL or fifteen weeks after CCl_4_-intoxication, rats received atorvastatin or control chow for 7 days before sacrifice. (**B**) Portal pressure was decreased in CCl_4_ rats and significantly upregulated in PPVL rats. (**C**) Hepatic vascular resistance was significantly downregulated in CCl_4_ as well as in PPVL rats. (**D**) Splanchnic vascular resistance remained unchanged in CCl_4_ rats and was significantly decreased in PPVL rats. (**E**) Shunt flow was decreased in CCl_4_ rats and significantly enhanced in PPVL rats after statin treatment compared to control. (**F**) Systemic vascular resistance was significantly reduced in PPVL rats and remained unchanged in CCl_4_-intoxicated rats. (**G**) In PPVL, as well as in CCl_4_-intoxicated rats, cardiac output was increased.

**Figure 3:**
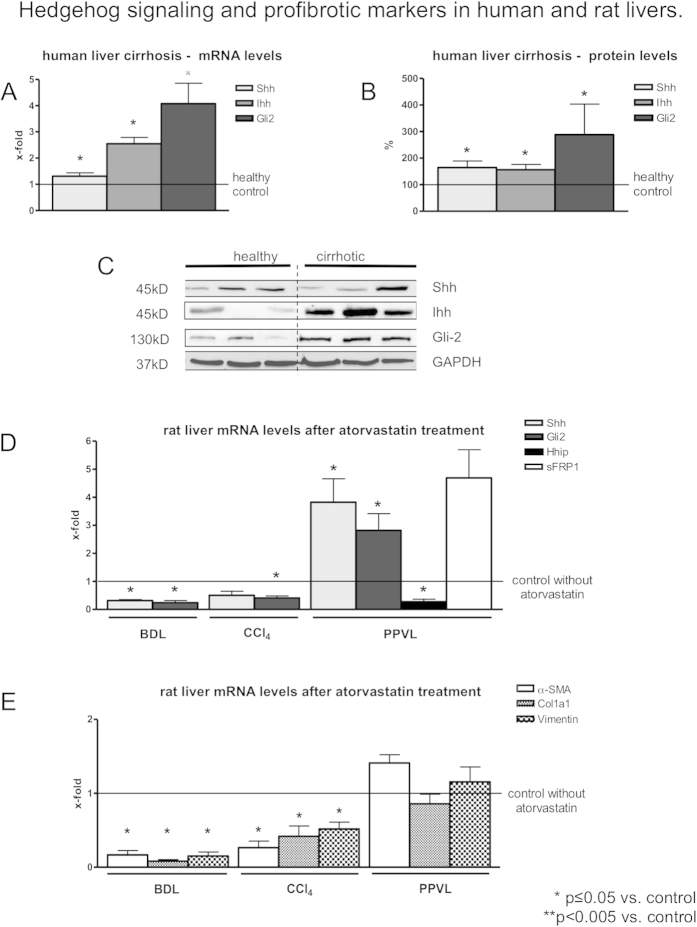
Hedgehog-signaling and profibrotic markers in human and rat livers. (**A**) Shh and Gli-2 mRNA levels were upregulated in human cirrhotic liver samples and (**B**,**C**) protein expression levels of Shh and Gli-2 were significantly increased confirming previous data in animal models^19^,^20^,^21^,^22^,^23^,^24^,^25^, which were not repeated in this study. (**D**) Shh and Gli-2 mRNA levels were significantly downregulated in BDL and CCl_4_-intoxicated cirrhotic rat livers and significantly upregulated in PPVL liver samples after atorvastatin treatment. (**E**) The profibrotic markers α-SMA, Col1a1 and Vimentin were downregulated in BDL and CCl_4_-intoxicated rat livers after statin treatment and not affected in PPVL rat livers. In Fig. 3C the blots shown are from cropped nitrocellulose membranes, however the gel was not cropped and therefore have been run under the same experimental conditions. The cropping lines are shown in black.

**Figure 4:**
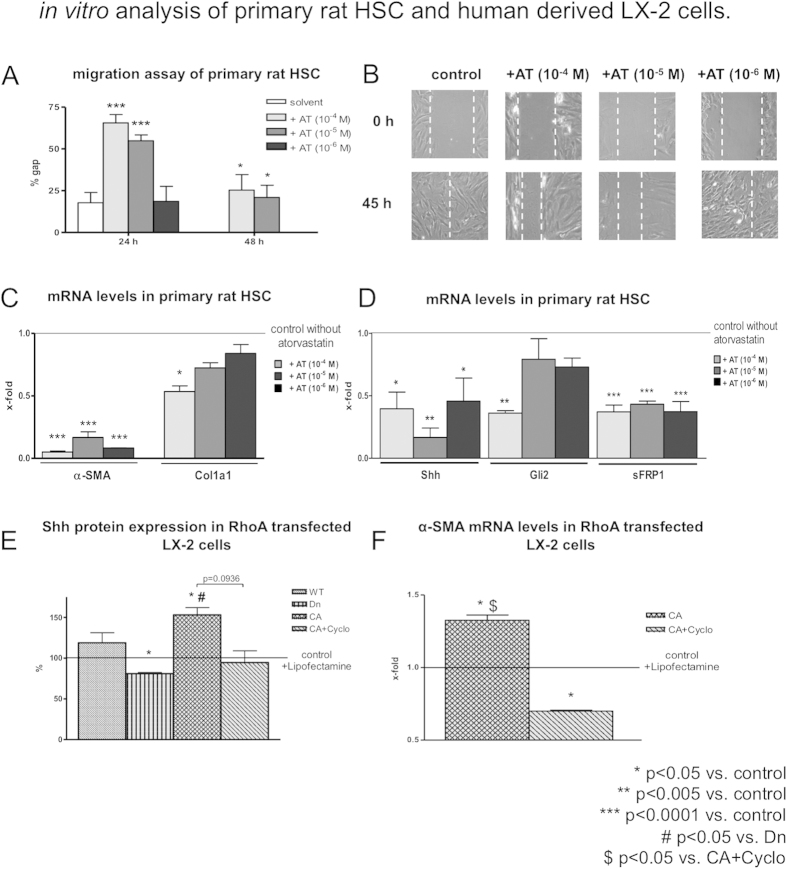
*In vitro* analysis of primary rat HSCs and human derived LX-2 cells. (**A**,**B**) HSCs had a reduced migration capability after incubation with atorvastatin. (**C**) α-SMA and Col1a1 mRNA levels were significantly reduced in HSCs incubated with atorvastatin. (**D**) Shh, Gli-2 and sFRP1 mRNA expression was downregulated in atorvastatin incubated HSCs. (**E**) Constitutive acitve (CA) RhoA increased Shh protein expression levels and cyclopamine inhibited this effect. (**F**) α-SMA mRNA levels in constitutive active RhoA transfected LX-2cells were upregulated and cyclopamine reversed this effect.

**Figure 5:**
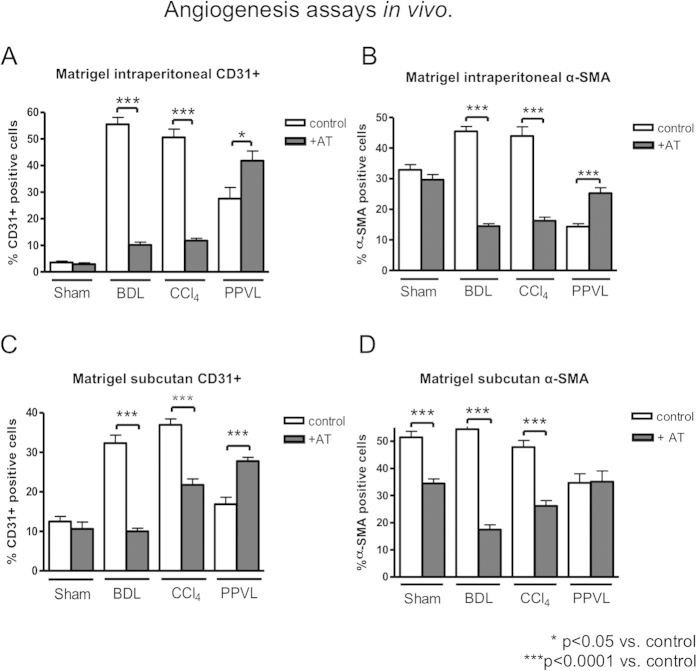
Angiogenesis assays *in vivo.* (**A**) CD31 positive cells were reduced in intraperitoneal implanted matrigels of BDL and CCl_4_-intoxicated rats and enhanced in PPVL rats. (**B**) In BDL and CCl_4_-intoxicated rats, α-SMA positive cells were decreased in intraperitoneal implanted matrigels and upregulated in PPVL rats. (**C**) CD31+ cells were decreased in subcutaneous implanted matrigels of BDL and CCl_4_-intoxicated rats after atorvastatin treatment and upregulated in PPVL rats. (**D**) After atorvastatin treatment the amount of α-SMA positive cells in subcutanous implanted matrigel was decreased in Sham, BDL and CCl_4_-intoxicated rats and remained unchanged in PPVL rats.

**Figure 6:**
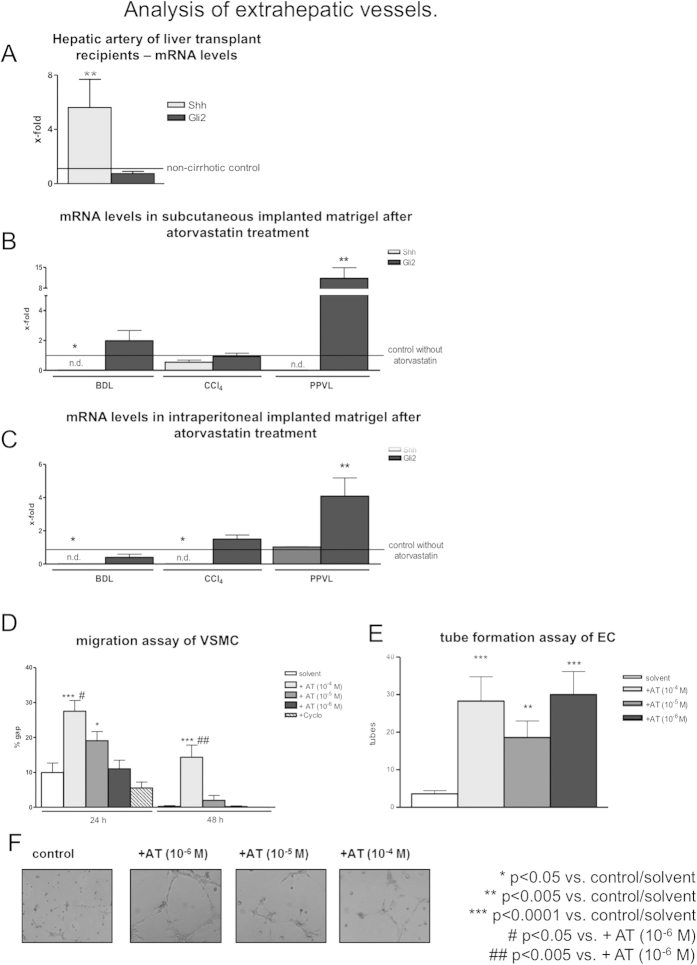
Analysis of extrahepatic vessels. (**A**) Shh mRNA levels were highly upregulated in hepatic arteries of liver transplant recipients compared to donors. (**B**) Shh mRNA levels were downregulated in subcutaneous implanted matrigel of BDL rats after atorvastatin treatment. Gli-2 was highly upregulated in subcutaneous implanted matrigels of PPVL rats. (**C**) Shh mRNA levels were reduced in intraperitoneal implanted matrigel of BDL and CCl_4_-intoxicated rats after statin treatment and Gli-2 was highly upregulated in PPVL rats. (**D**) Atorvastatin reduced migration of A7r5 rat aortic VSMCs significantly *in vitro*. Incubation with cyclopamine had no effect on migration capability of VSMCs. (**E**,**F**) Endothelial cells (HUVEC) formed more tubes after incubation with atorvastatin in tube formation assay.

In summary, these data suggest a greater role of Shh/RhoA-signaling in the extrahepatic angiogenesis in cirrhosis, whereas Gli-2 is a predominant mediator of canonical Hh-pathway in non-cirrhotic portal hypertension (Fig. 1B).

## Discussion

This study shows for the first time, that the mechanisms of neo-angiogenesis in cirrhotic and non-cirrhotic portal hypertension are different. Statin therapy blunts neo-angiogenesis in liver cirrhosis, probably due to an inhibition of non-canonical Hedgehog signaling among other mechanisms. Furthermore, in non-cirrhotic portal hypertension, statins increase splanchnic and systemic angiogenesis, together with an upregulation of the canonical Hedgehog pathway activity.

In previous studies we showed that atorvastatin significantly blunts portal hypertension and fibrogenesis in BDL rats^13^,^14^, and that statins inhibit proliferation and apoptosis and induce senescence in hepatic myofibroblasts^15^. Our present study confirms these results in a second model of fibrosis (CCl_4_-intoxication): also in the CCl_4_-induced cirrhosis, the profibrotic markers α-SMA and collagen-1 were decreased after statin treatment.

Hedgehog pathway activation has previously been identified as a cause of activation of hepatic stellate cells and in consequence contraction and production of extracellular matrix^29^. Indeed, the present study clearly shows an upregulation of the Hedgehog pathway, associated with progression of liver fibrosis. Moreover, statin treatment reduced Hedgehog pathway activity in cirrhotic livers and in activated hepatic stellate cells. This might be a consequence of the previously suggested non-canonical mechanisms of Hedgehog downstream activation, via RhoA/Rho-kinase^17^,^30^, since atorvastatin inhibits RhoA^13^,^14^, which is clearly interacting with Hh-signaling as shown by our gain of function experiments in HSCs. At least partly by this mechanism, statins decrease Hedgehog pathway activity in cirrhotic rat livers. Moreover, statin treatment may lead to inhibition of canonical Hedgehog pathway, shown by decreased Gli-2 expression. This probably is a further explanation for the previously described beneficial effects of statins on liver fibrosis^13^,^14^,^15^.

In cirrhotic BDL and CCl_4_-intoxicated rats not only fibrosis was blunted by statins, but also the intrahepatic vasoconstriction was significantly reduced^13^,^31^. In line with these previously published findings from others and our group, a significant decrease in portal pressure in BDL rats and a trend towards lower portal pressure in CCl_4_-intoxicated rats after treatment with atorvastatin was reported^13^,^31^. This difference between the models might be caused by a more aggressive, cholestatic injury induced by the BDL model.

Portal hypertension leads to increased neo-angiogenesis and thereby enhances portal-systemic shunting^3^. Our current study not only confirmed previous finding in BDL rats that the hepatic shunt flow was reduced in atorvastatin-treated rats^13^, but also extended this finding to the CCl_4_-model. Summarizing previous and current results, atorvastatin treatment decreased fibrogenesis, hepatic resistance and shunt flow in both cirrhotic models in rats.

Moreover, statins reduced neo-angiogenesis in the splanchnic circulation. The decreased splanchnic neo-vascularization in statin-treated cirrhotic animals may be due to the decreasing effects of atorvastatin on fibrosis and portal pressure. Thereby, shear stress in splanchnic vessels is reduced and one of the major driving forces for angiogenesis in portal hypertension is blunted. However, atorvastatin blunted the neo-vascularization of subcutaneously implanted matrigels in the cirrhotic rats. Therefore, these results cannot only be explained by the role of portal pressure, but also by the general anti-angiogenic effect of statins, that might be mediated by Hedgehog signaling. Indeed, Hedgehog pathway is crucially involved in angiogenesis, tumor vascularization and remodeling in diseased livers^32^,^33^,^34^. The reduced Hedgehog pathway activity in cirrhotic livers may affect the splanchnic angiogenesis via influencing endothelial and vascular smooth muscle cells. Interestingly, and similar to the findings in rats, in human cirrhosis the Hedgehog ligands, but not their effector Gli-2, are upregulated. Therefore, a possible explanation could be the inhibition of circulating pro-angiogenetic factors, as well as Hedgehog components, which in cirrhosis generally show increased expression and are released into the circulation.

Statin treatment of non-cirrhotic portal hypertensive rats led to surprising and contrary results. In these rats atorvastatin increased portal pressure and shunt flow, probably due to highly upregulated neo-angiogenesis in peritoneal and subcutaneous vessels. Contrary to its effect in hepatic and cirrhotic vessels, statin treatment enhanced the activation of Hedgehog pathway in splanchnic and systemic vessels of PPVL rats. Fibrosis, inflammation and portal hypertension in cirrhosis might lead through different cytokines and chemokines to extrahepatic vascular changes. Since atorvastatin ameliorates fibrosis and portal hypertension, these liver derived effects are less pronounced after atorvastatin treatment. In PPVL the liver is not cirrhotic and the hepatic effects of statins mediated by modulation of cirrhosis are lacking. This might be a possible explanation, that atorvastatin has opposing effects in PPVL rats due to a non-canonical Hedgehog regulation. Furthermore, this study showed that different molecular mechanisms underlie angiogenesis in cirrhotic and non-cirrhotic portal hypertension.

In conclusion, the broad use of statins, and their recently recognized beneficial effect on liver cirrhosis and portal hypertension^9^,^35^,^36^ point towards a potential clinical relevance of this study. While our data present further evidence for the beneficial use of statins in liver cirrhosis, caution is required in non-cirrhotic portal hypertension. Further studies are warranted, to investigate the different cellular mechanisms regulating angiogenesis in cirrhotic and non-cirrhotic portal hypertension.

## Materials and Methods

### Animals

55 wild type (WT) rats (Sprague Dawley) were used for our experiments. All experiments were performed in accordance with relevant guidelines and regulations approved by LANUV, the responsible committee for animal studies in North Rhine-Westphalia (permission number 8.87-50.10.31.08.287). Sprague Dawley rats were housed in a controlled environment (12 hour light/dark, temperature 22 °C to 24 °C), and fed standard rat chow (Ssniff, Soest, Germany).

### Model of cirrhotic portal hypertension

#### Cholestatic model of cirrhosis

Bile duct ligation (BDL) was performed in nine WT rats with an initial body weight (BW) of 180–200 g as described previously^13^,^37^. Experiments were carried out six weeks after BDL. Eight sham-operated rats served as controls. Five rats undergoing BDL for six weeks received atorvastatin (15 mg/kg BW per day) on the last seven days before sacrifice, as described previously^13^.

#### Toxic model of cirrhosis

20 rats with an initial BW of 100–120 g twice weekly underwent inhalation of 1l/min CCl_4_ for 16 weeks until ascites, as a definite sign of portal hypertension, was present as described previously^37^. Seven rats undergoing CCl_4_-intoxication for 16 weeks received atorvastatin (15 mg/kg BW per day) on the last seven days before sacrifice.

### Model of non-cirrhotic portal hypertension

#### Partial portal vein ligation (PPVL)

PPVL was performed in 18 WT rats with an initial BW of 180–200 g as described previously^38^,^39^. Experiments were carried out two weeks after PPVL. Seven rats undergoing PPVL for two weeks received atorvastatin (15 mg/kg BW per day) on the last seven days before sacrifice^38^,^39^.

#### *In vivo* hemodynamic experiments

*In vivo* studies were performed in eleven WT PPVL rats, 13 WT CCl_4_ and nine WT BDL rats and their respective controls. Rats were used for the hemodynamic studies as described previously^40^. To assess the effects of atorvastatin, invasive measurements of mean arterial pressure (MAP) and portal pressure (PP) were performed in cirrhotic rats.

#### Microsphere technique

To investigate hemodynamics, the colored microsphere technique was performed as described previously^40^. 300.000 systemic (red) microspheres (15 μm diameter, Triton-Technologies, San Diego, USA) were injected in the left ventricle. Mesenteric portal-systemic shunt volume was estimated by injection of 150.000 microspheres (yellow) in the ileocecal vein^40^.

#### Human liver samples

The Human Ethics Committee of the University of Bonn (202/01) approved the use of human liver and hepatic arteries, taken during liver transplantation from patients with alcohol-induced cirrhosis (n = 5). Liver and hepatic artery samples from non-cirrhotic patients submitted to liver resection served as controls (n = 5). The methods were carried out in accordance with the approved guidelines. An informed consent was obtained from all patients. No patient or donor received catecholamines, ACE inhibitors or angiotensin receptor antagonists before transplantation^37^. Samples were snap frozen following excision.

#### Western blotting

Liver samples were processed as previously described using SDS-PAGE gels and nitrocellulose membranes^13^,^15^. Ponceau-S staining assured equal protein loading. GAPDH served as endogenous controls. Membranes were incubated with the respective primary antibody (Supplementary Table 1) and corresponding secondary peroxidase-coupled antibody (Santa-Cruz-Biotechnology, Santa Cruz, USA). After enhanced chemiluminescence (ECL, Amersham, UK) digital detection was evaluated using Chemi-Smart (PeqLab Biotechnologies, Erlangen, Germany).

#### Quantitative RT-PCR

RNA isolation, reverse transcription and detection by RT-PCR were performed as described previously^13^,^37^. Assays provided by Applied Biosystems (Foster City, USA) are listed in Supplementary Table 2. 18S rRNA served as endogenous control. Results of HSCs and liver samples were expressed as 2^−ΔΔC^_T_, and express the x-fold increase of gene expression compared to the control group.

#### Immunohistochemical staining for CD31 and α-SMA

Stainings for CD31 and α-SMA were performed in cryosections from liver tissue (3 and 7 μm thick, respectively) as described previously^41^,^42^,^43^. Briefly, after several steps cryosections were incubated with a mouse-anti-SMA antibody (clone 1A4; Sigma-Aldrich, Munich, Germany), or with antibody against CD31 (ab24590, Abcam, Cambridge, UK). Thereafter, a biotinylated rabbit-anti-mouse secondary antibody (Dako, Glostrup, Denmark) was used.

#### Isolation of primary HSCs

Rat HSCs were isolated as described previously^37^,^44^. Briefly, primary HSCs were isolated in a two-step pronase-collagenase perfusion from the livers of WT rats and fractionated by density gradient centrifugation. Viability and purity were systematically over 95%. Cells were seeded on uncoated plastic culture dishes. Experiments were performed seven days after isolation or after the first passage (10 days) when HSCs were fully activated.

#### Incubation with atorvastatin

Atorvastatin (10^−6^ M, 10^−5^ M, 10^−4^ M) was added to the culture medium of these cells as indicated for three days, or cells remained untreated.

#### Transfection with RhoA plasmids

LX-2 cells were kindly provided by our co-operation partner Vijay H. Shah (Mayo Clinic, Rochester, USA)^45^. Twenty-four hours before transfection, 6 × 10^5^ LX2 cells were incubated with transfection media (Dulbecco’s modified Eagle’s medium [DMEM] with 10% fetal calf serum [FCS] without penicillin/streptomycin). The RhoA plasmid (pEGFP-C2) and the respective empty control plasmid were isolated according to manufacturer instructions (NucleoBond Xtra Maxi kit; Machery, Nagel, Germany). Plasmid (15 μg) and 37.5 μL of lipofectamine were incubated for 20 minutes with a total volume of 3.6 mL of media. After removal of media the plasmid/lipofectamine mix was added drop-wise to cells. After 3–4 hours, mixture removed and cells were again incubated with fresh media, containing 10% FCS, and harvested after 3 days. Efficacy of transfection was tested by their GFP-expression and confirmed by RT-PCR and Western blotting.

#### Scratch assays

To observe the cell migration a scratch assay was performed as described previously^28^. Briefly, primary rat HSCs or A7r5 VSMCs were cultured in 24-well plates until the wells were completely covered with cells. A line was scratched through the cell layer with a pipette tip (1 mm). Directly after scratching and at defined time points pictures were taken (Nikon, Eclipse 50i) and the scratch width was measured using the software NIS Elements D 3.2 (Zeiss, Germany).

#### Tube formation

To evaluate *in vitro* effects of atorvastatin on endothelial cells (HUVEC) (PromoCell, Heidelberg, Germany), a tube formation assay was performed as described previously^46^,^47^. A 24-well plate was coated with 300 μl Matrigel (Gibco/BRL, Karlsruhe, Germany) containing 10 ng/ml VEGF. After 24 h, 2.5 × 10^4^ endothelial cells in 75 μl medium (Endothelial Cell Growth Medium+Supplemental Mix, PromoCell, Heidelberg, Cat.No. C-22010) were seeded in each well. Intercellular connections and tube-like formations were counted using a Nikon, Eclipse 50i microscope at 100× magnification.

#### Statistical analysis

Results are expressed as mean ± SEM unless otherwise indicated. Data were analyzed using Student’s unpaired and paired t-test where appropriate. Statistical analyses and graphing were performed using GraphPad Prism 4.0 for Macintosh. P < 0.05 was considered statistically significant.

## Additional Information

**How to cite this article**: Uschner, F. E. *et al.* Statins activate the canonical hedgehog-signaling and aggravate non-cirrhotic portal hypertension, but inhibit the non-canonical hedgehog signaling and cirrhotic portal hypertension. *Sci. Rep.* **5**, 14573; doi: 10.1038/srep14573 (2015).

## Supplementary Material

Supplementary Information
