# Supplementary material for: Statins activate the canonical hedgehog-signaling and aggravate non-cirrhotic portal hypertension, but inhibit the non-canonical hedgehog signaling and cirrhotic portal hypertension
Source: Sci Rep. 2015 Sep 28;5:14573. doi: 10.1038/srep14573 (PMC4585958; doi:10.1038/srep14573)

**Statins activate the canonical hedgehog-signaling and aggravate non-cirrhotic portal hypertension, but inhibit the non-canonical hedgehog signaling and cirrhotic portal hypertension.**

**Authors:**

Frank E. Uschner¹†, Ganesh Ranabhat¹†, Steve S. Choi^2^, Michaela Granzow^1^, Sabine Klein¹, Robert Schierwagen¹, Esther Raskopf¹, Sebastian Gautsch¹, Peter F.M. van der Ven^3^, Dieter O. Fürst^3^, Christian P. Strassburg^1^, Tilman Sauerbruch¹, Anna Mae Diehl^2^, Jonel Trebicka¹*

† contributed equally

* Corresponding author

**Affiliations:**

¹Department of Internal Medicine I, University of Bonn, Germany.

^2^Division of Gastroenterology, Department of Medicine, Duke University Medical Center, Durham, North Carolina, USA.

^3^Institute for Cell Biology, University of Bonn, Germany.

Corresponding author: Jonel Trebicka, Department of Internal Medicine I, University of Bonn, Sigmund-Freud Str. 25, D-53105 Bonn, Germany. [jonel.trebicka@ukb.uni-bonn.de](mailto:jonel.trebicka@ukb.uni-bonn.de), Tel: +49 228 287 15507, Fax: +49 228 287 19718

**Supplemental Table 1:** List of primary antibodies used for detection of protein expression levels by Western blots.

**Supplemental Table 2:** Shh, Ihh, Gli-2, α-SMA, Col1a1 and Vimentin were detected by RT-PCR with the listed probes.

**Supplemental Figure 1:** **VSMC and EC migration *in vivo.*** (A) Intraperitoneal migration of CD31+ positive cells was enhanced in BDL, CCl_4_ and PPVL compared to sham-operated rats. Atorvastatin reduced CD31+ cell migration in BDL and CCl_4_ rats, whereas it was enhanced in PPVL rats. (B) Migration of α-SMA positive was enhanced in BDL and CCl_4_ compared to sham-operated rats and decreased by atorvastatin treatment. In untreated PPVL rats, migration of α-SMA positive cells was lower than in sham-operated rats, but increased after atorvastatin treatment. (C) Atorvastatin treatment reduced CD31+ cell migration in subcutan matrigel of BDL and CCl_4_ rats, whereas it enhanced migration in PPVL rats. (D) In BDL and CCl_4_ rats, atorvastatin treatment reduced α-SMA positive cell migration. In PPVL rats, atorvastatin had no significant effect on migration of α-SMA positive cells.


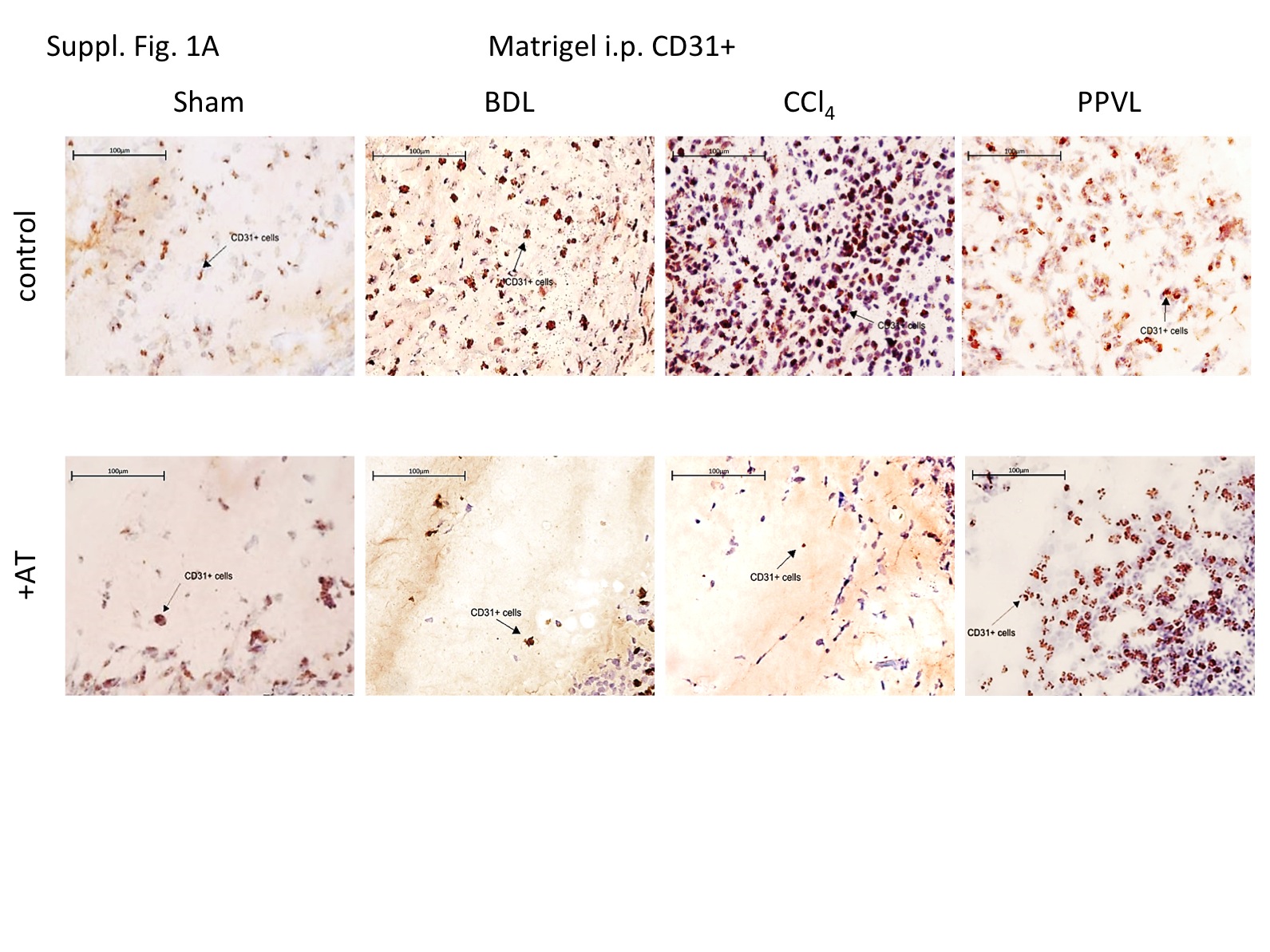

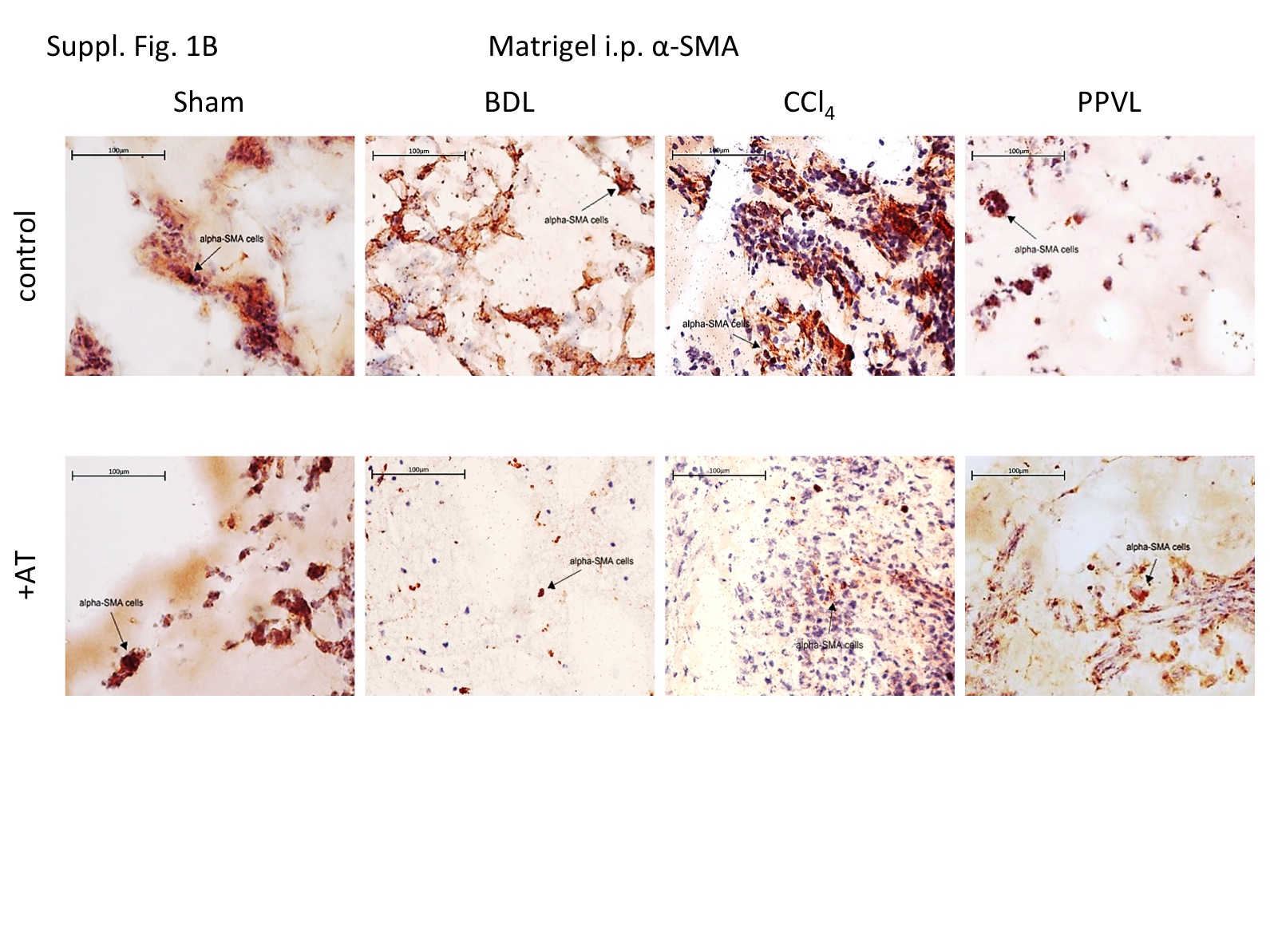


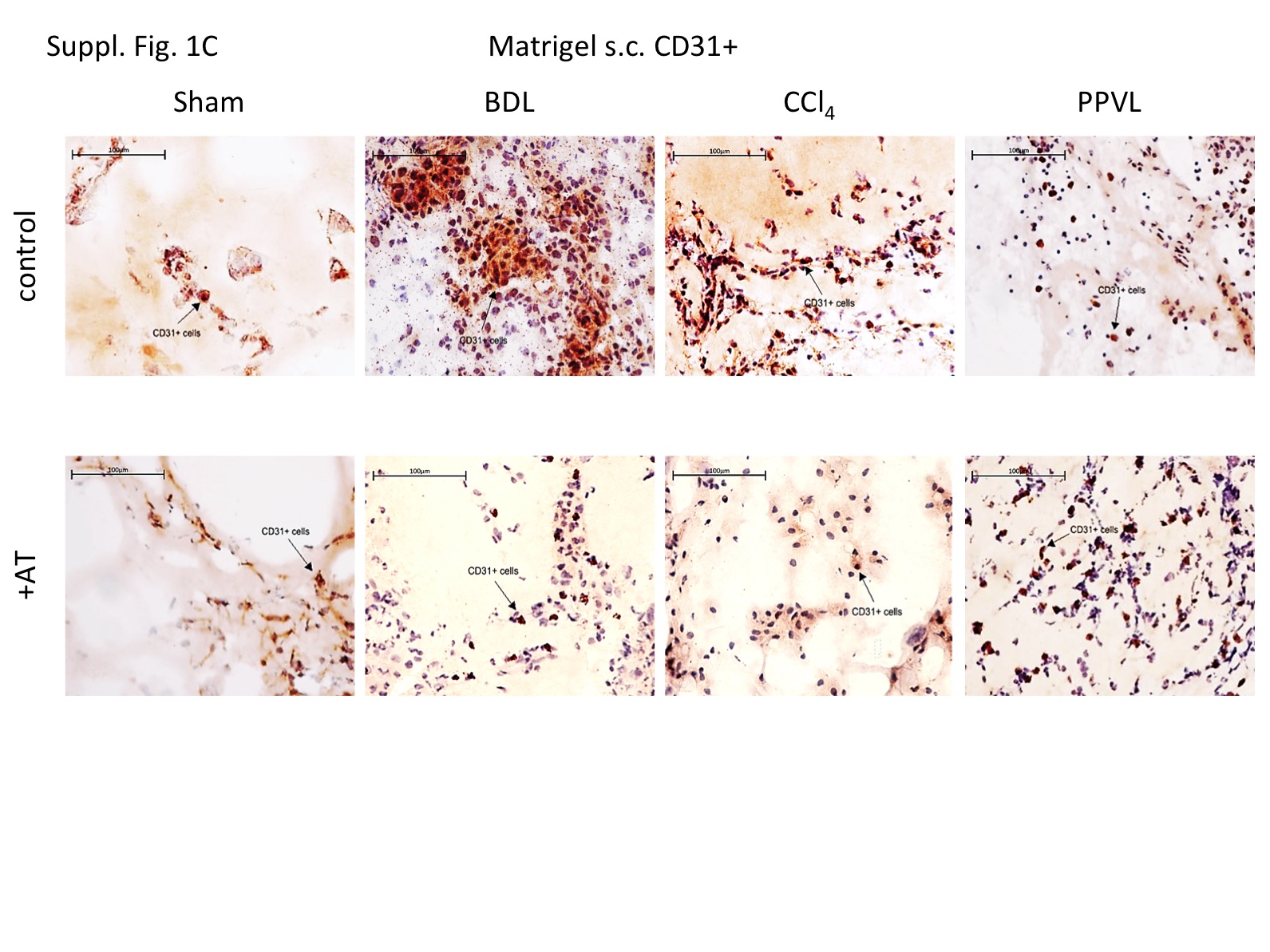

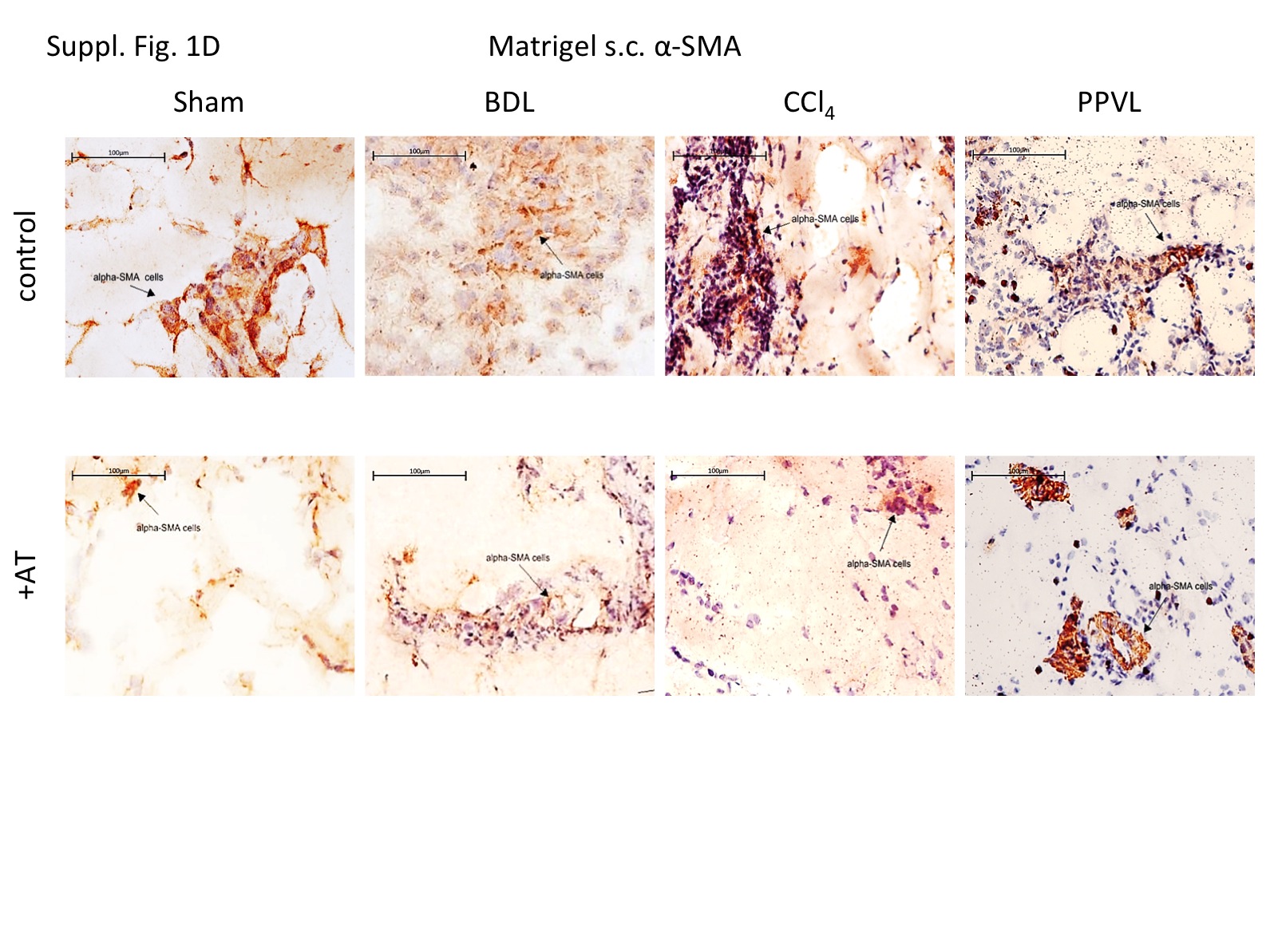

Supplement: Supplementary Information [file srep14573-s1.docx]
